# Chikungunya Virus Glycoproteins Pseudotype with Lentiviral Vectors and Reveal a Broad Spectrum of Cellular Tropism

**DOI:** 10.1371/journal.pone.0110893

**Published:** 2014-10-21

**Authors:** Dakang Hu, Jin Zhang, Hua Wang, Shuangchun Liu, Lianhua Yu, Lingfen Sun, Ying Qu

**Affiliations:** 1 Department of Laboratory Medicine, Taizhou Municipal Hospital, Taizhou, China; 2 Institute of Infectious Diseases, Union Hospital, Tongji Medical Collage, Huazhong University of Science and Technology, Hubei Wuhan, China; SRI International, United States of America

## Abstract

**Background:**

Outbreaks of the Chikungunya virus (CHIKV) infection has been documented in over 40 countries, resulting in clinical symptoms characterized by fever and joint pain. Diagnosing CHIKV in a clinical lab setting is often omitted because of the high lab safety requirement. An infection system that mimics CHIKV infection will permit clinical evaluation of the production of neutralizing antibody for both disease diagnostics and treatment.

**Methodology/Principal Findings:**

We generated a CHIKV construct expressing CHIKV structural proteins. This construct permits the production of CHIKV pseudo-viral particles with a luciferase reporter. The pseudo-virus was able to infect a wide range of cell lines. The pseudovirus could be neutralized by the addition of neutralizing antibodies from patients.

**Conclusions:**

Taken together, we have developed a powerful system that can be handled at biosafety level 2 laboratories for evaluation of existence of CHIKV neutralizing antibodies.

## Introduction

The Chikungunya virus commonly referred to as CHIKV is a word derived from the Kimakonde language meaning ‘walking bent over’ [1]. Infected individuals often assume a bent posture due to an inflammatory response in their joints and find it hard to maneuver their limbs (almost to a point of paralysis). Outbreaks of CHIKV have been documented as early as 1779 and frequent outbreaks have been reported through 1960–2003 in areas of South and Southeast Asia. The most notable outbreak was seen in French Reunion Island through 2005 and 2006 where about one-third of the entire country's population was infected by CHIKV. Out of a total population of 785,000 people 300,000 cases were reported including a total of 237 deaths. CHIKV has currently been documented in over 40 countries and is listed as a US National Institute of Allergy and Infectious Diseases (NIAID) as a category C priority pathogen [1]. An outbreak of CHIKV infection was also reported in Guangdong province, China in 2010 [2].

CHIKV belongs to the genus of Alphavirus of Togaviridae family. It has a single stranded RNA genome approximately 11.8 Kb in length. Infectious virions adopt an icosahedral shape with a 60–70 nm capsid, and a phospholipid envelope [3], [4]. CHIKV genome contains two open reading frames (ORFs), a 5′ cap structure and a 3′ poly A tail. The first ORF is responsible for producing the non-structural proteins (NS) with two polyprotein precursors of NSP1, NSP2, NSP3, and NSP4. The second ORF encodes the viral structural proteins: the capsid proteins; envelope glycoproteins E1, E2, and E3 and an additional protein, 6K. E1 may be responsible for promoting the release of the viral nucleocapsid [5]. Envelope protein E2 is postulated to be responsible for viral attachment. The viral envelope protein E3 appears to protect E1 from fusogenic conformational changes during egress, and is a secreted protein. A report indicated that there are two-hundred-and-forty copies of E1 and E2 forming heterodimers that are studded into the viral membrane [6], [7]. Together these glycoproteins would appear to be the main drivers in attachment to the host cell. The E2 and E1 heterodimers have been shown to cause viral membrane fusion by a cholesterol dependent mechanism [5]. The 6K (approximately 6000 Da) protein appears to potentially have multiple roles in glycoprotein processing, cell permeabilization, and viral budding [5], [8], [9], [10], [11].

A major hurdle for clinical laboratories to work with CHIKV is that the virus is typically studied at Biosafety Level 3 (BSL-3). To many small laboratories in developed countries, the required laboratory setting is prohibiting. On the other hand, pseudoviruses that can be handled in a BSL 2+ laboratory would be useful and allow evaluation of the production of neutralizing antibodies. To this end, we reported the successful development of a pseudo-viral system which resembles CHIKV infection without expressing its non-structural proteins. The pseudo-virus was able to infect many cell lines. Most importantly, this pseudovirus can be neutralized by sera derived from CHIKV infected individuals. Taken together, we have developed a robust CHIKV infection system that can be used in clinical evaluation of virus infection and production of neutralizing antibodies.

## Results

### Construction of CHIKV Structural Proteins

**Figure 1:**
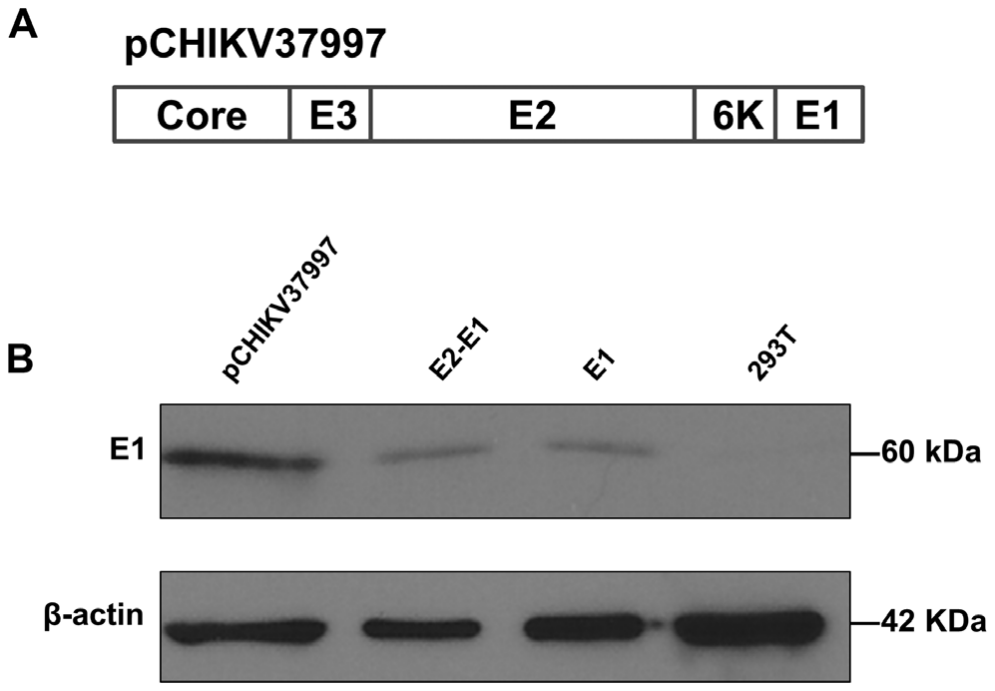
Expression of CHIKV glycoproteins. **A**. Structural proteins in the pCHIKV37997 construct. **B**. 10^5^ 293T cells were transfected with pCHIKV37997 or plasmids expressing CHIKV E1 or EIE2. Total protein was harvested at 2 days post-transfection and subjected to western blotting analysis using the rabbit anti-CHIKV E1 serum.

To express CHIKV envelope proteins, we obtained DNA sequences of relevant region of the early African CHIKV strain 37997 for gene synthesis. The derived construct (pCHIKV 37997) contained the CHIKV viral structural proteins from the capsid to the E1 protein (Figure 1A). After transfection into 293T cells, we were able to detect the expression of E1 from this construct (Figure 1B). For subsequent experiments, we also subcloned E1, E2, and E1E2 into separate DNA plasmids.

### Production of CHIKV pseudovirus (CHIKVpp)

**Figure 2:**
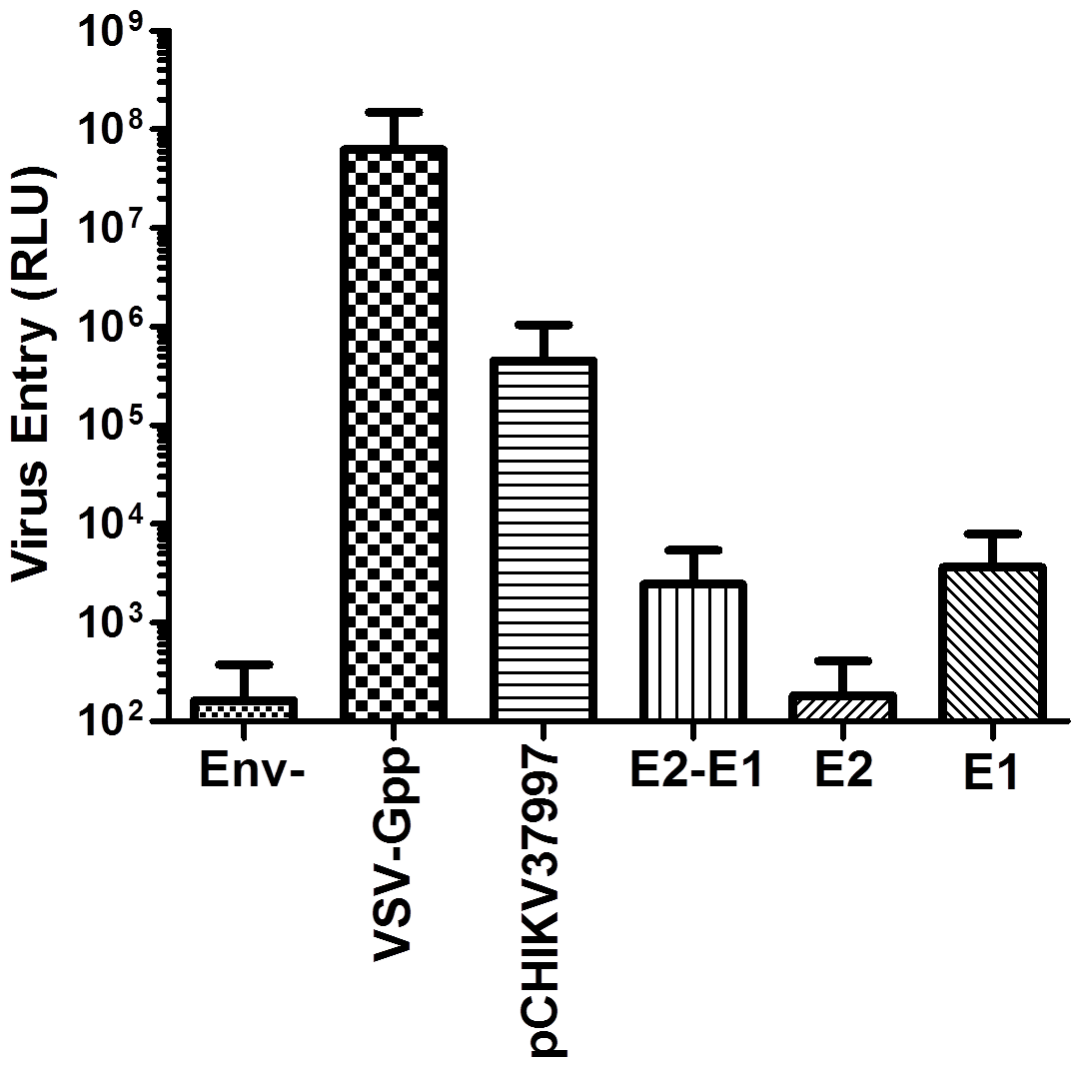
CHIKVpp infection of 293T cells. 10^5^ 293T cells transfected with lentiviral vector and packaging construct with CHIKV37997, or VSV-G, or E1, E2, or E1E2 expression plasmid. Two days post-transfection, supernatants were collected and added to naïve 293T cells for three hours at 37°C. Infected cells were incubated for an additional 48 hours prior to luciferase assay (n = 3, mean± S.D.).

To produce pseudovirus, we transfected 293T with pCHIKV 37997 and a lentiviral construct carrying firefly luciferase gene and the lentivirus packaging construct. 48 hrs post-transfection, we collected virus from the supernatants. To control for specificity, we also similarly packaged pseudovirus using CHIKV E1, or E2, or E1E2 only, or an empty pcDNA3 vector (Env-). We then infected native 293T cells with different pseudoviruses and it was found that only the full length CHIKV construct (pCHIKV 37997) carrying all structure proteins was able to yield infectious virus (Figure 2). Our findings are consistent with what has been reported (Salvador et al). Overall, this data confirmed that lentiviral vectors can pseudotype with CHIKV envelope proteins into infectious virus. Derived pseudovirus is infectious for only a single round and can be safely handled in a BSL-2 laboratory.

### CHIKVpp infected a wide range of cells in vitro

Subsequently, we tested the cellular tropism of CHIKVpp. Among the cell lines that we tested, CHIKVpp displayed considerable infectivity on most adherent cells (Table 1). By contrast, CHIKVpp failed to infect those lymphocyte-originated cell lines. Moreover, CHIKVpp was unable to infect the mosquito derived C6/36 cell line, which is likely due to the inefficient replication and production of firefly luciferase from the lentiviral vector in the insect cell line.

**Table 1:** Cellular permissivity measured by CHIKV pseudovirus infection.

Cell Line	Infectable (+) uninfectable (−)	Relative Permissiveness (RLUs)
HEK293T	+	*****
HeLa	+	*****
Caco2	+	***
HBMEC	+	*****
C6/36	−	*
Raw264.7	±	**
J774	+	**
HUVEC	+	****
HepG2	+	*****
Jurkat T cell	−	*
H9 human T cell line	−	*
CEM human T cell line	−	*

Relative light units (RLU) are shown as averages of three independent experiments.

* Under 200 RLUs,

** 1000–10,000 RLUs,

*** 10,001–20,000 RLUs,

**** 50,000–1,000,000 RLUs,

***** 1,000,001–2,000,000 RLUs.

Env- pseudovirus infection yielded RLUs under 200RLUs and were considered negative in Column 2.

**Figure 3:**
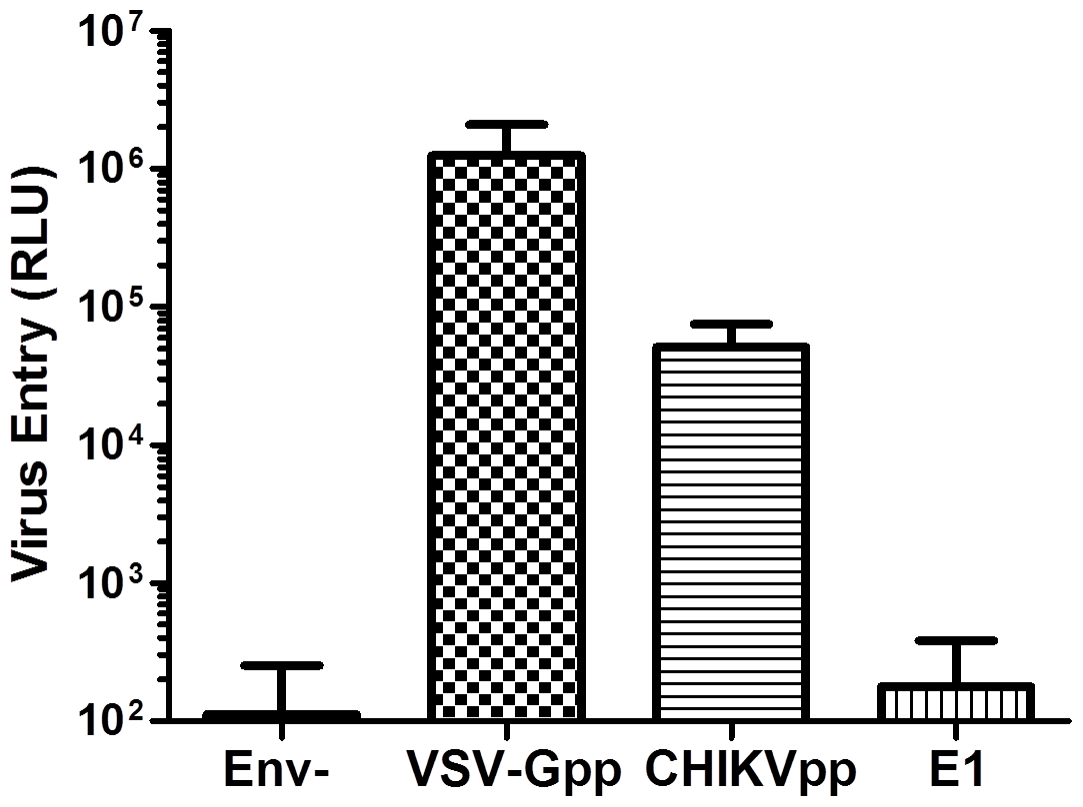
CHIKVpp infection of joint cells. 5×10^5^ cells isolated from human joint tissues were infected by CHIKVpp or VSV-Gpp. Luciferase assay was determined two days post-infection.

To test if CHIKVpp infects joint cells, we obtained human joint tissues and prepared cells accordingly. Strikingly, we were able to achieve robust infection of the primary cells by CHIKVpp (Figure 3). This data is significant because it indicates that CHIKVpp and the cells isolated from joints may be a model system to study the tissue infection by this virus.

### Neutralization of CHIKVpp by patients-derived antibodies

**Figure 4:**
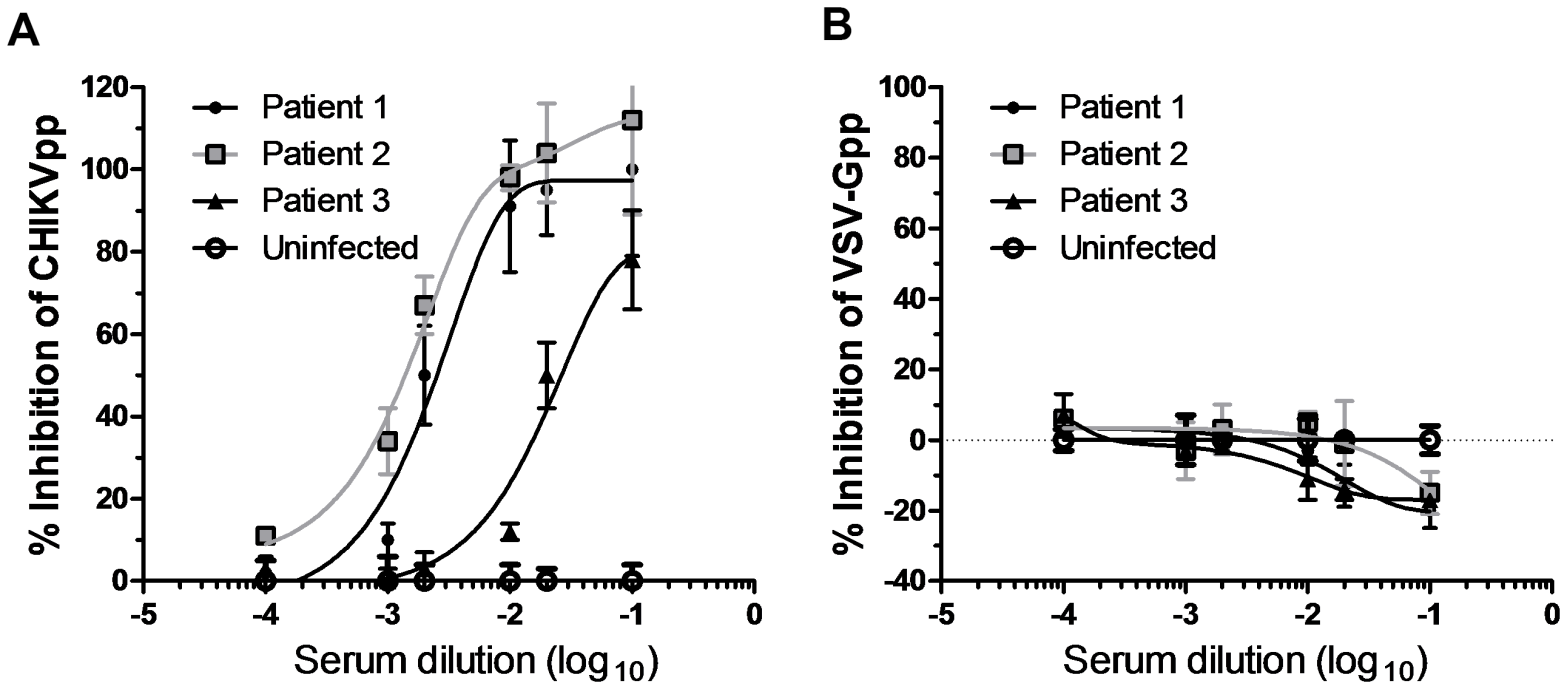
Neutralization of the CHIKV-pseudovirus by patients' derived anti-sera. **A**. Dose-dependent neutralization of CHIKV-pseudovirus by serum samples from three CHIKV infected patients. Normal serum from a healthy donor was included as negative control. Luciferase assay was determined two days after infection. **B**. VSV-G pseudovirus infection was not blocked by the three patients' sera. Pseudoviruses were incubated with serially diluted serum. The mixture was added to cells and luciferase activity was measured. The percent inhibition by CHIKV-infected patients sera (no. 1–3) was calculated relative to normal serum from an uninfected donor (n = 3, mean± S.D.).

One of the most easily noticed applications of CHIKVpp is the detection of neutralizing antibodies in infected individuals for clinical diagnosis or prognosis. For this purpose, we collected serum samples from three CHIKV infected individuals. Addition of anti-sera to CHIKVpp indeed blocked infection in a concentration-dependent manner (Figure 4). By contrast, serum from healthy individual was unable to inhibit CHIKVpp infection.

## Discussion

Chikungunya virus has increasingly drawn public attention after causing many detrimental results at Reunion Island. For clinical researchers, a major hurdle in working with CHIKV is the relatively high standard on laboratory safety setting. It is inconvenient for a local clinic to thoroughly evaluate the infection without a certified BSL-3 facility. We started out to tackle this problem by creating pseudovirus that bears CHIKV envelope proteins which recapitulates CHIKV entry. Such an experiment system allows convenient evaluation of the presence of CHIKV neutralizing antibodies. It also allows the study of CHIKV entry. Our method is similar to what has been reported [12], [13], [14].

Our current research has shown that the Chikungunya virus was infectious to numerous human cells. Interestingly the virus was shown to infect a blood brain barrier mimic, HBMECs. As to our knowledge there has not been an account previously published showing a correlation to patient symptoms associated with brain infection (such as headache, dizziness, etc). The infection of the blood brain barrier (although not showing an infection of actual brain cells) could provide more information on the possibility of CHIKV in causing infectivity in the brain. Previously it was thought that the Reunion Island outbreak spread so fast that a possible air borne mechanism could exist [1]. The infection of lung epithelial cell line A549 supports this speculation, although one article did show a contradiction to A549 infectivity [1].

Humans have been noted as the main reservoirs for the CHIKV virus, although many other animals have been noted to be reservoirs besides humans. The infection of the VERO African green monkey cells would show that other species of mammals are in fact able to be infected besides humans. This would provide more evidence on the basis that monkeys can be reservoirs for CHIKV. The only human cell line that seemed immune from CHIKV infection that was tested would appear to be lymphocyte-originated cell lines. It is conceivable that such non-permissive cell lines may be used in the future for the identification of putative entry factor that upon expression renders non-permissive cells permissive to CHIKV infection.

Besides infecting cell lines, CHIKV pseudo-virus was also able to infect primary cells isolated from joint tissues. This is exciting given that CHIKV infection often causes debilitating joint pains. Such a cellular model system will undoubtedly benefit future research that targets to identify joint-specific entry factors, and/or isolate specific join cell population that is prone to CHIKV infection.

A major application of CHIKVpp in clinics is to evaluate the production of neutralizing antibodies, which is important to both diseases diagnosis and prognosis. The described CHIKVpp represents a convenient experimental system that can be used to determine the titer of neutralizing antibodies. Due to the use of split plasmids, such a system can be readily set up in most clinical laboratories.

## Materials and Methods

### Cell Lines

All cell lines were purchased from ATCC and maintained in DMEM supplemented with 5% penicillin and streptomycin, 1% NEAA, and 10% fetal bovine serum (Hyclone) unless specified otherwise. C6/36 cells were maintained at 28°C, whereas all other lines were cultured in a 37°C incubator. Human epithelial kidney 293T (HEK 293T) cells, HeLa, human brain microvascular endothelial cells (HBMECs) (Purchased from Promo Cell), HepG2, HUVEC, A549 adenocarcinomic human alveolar basal epithelial cells, Caco-2 human epithelial colorectal adenocarcinoma cells, RAW264.7 and J774 mouse macrophage cell lines, Jurkat T lymphocyte cells, CEM-SS T4-lyphoblast cells, and C6/36 mosquito cells (derived from Ae.Albopictus mosquito larval tissue) were all of the cell types infected with the CHIKV pseudo-virus. Isolation of synovial cells was done following a published protocol from patients seeking surgery with knee problems [15]. Derived cells were maintained in Eagle's basal medium with 20% fetal calf serum). The joint cells were isolated from patients' joint tissue lavage by physicians and de-identified and provided to authors. Taizhou Municipal Hospital Review Board waived the need for consent and approved this study.

### DNA constructs

The structure region of CHIKV 37997 (Genebank Accession No. AY726732, nt 7569–11315) was used for gene synthesis (Genscript) and subcloned into pcDNA 3 vector. CHIKV E1 and E1E2 were subcloned between the BamH I and Xho I sites. The derived plasmid was named pCHIKV 37997. Lentiviral plasmid carrying luciferase gene, the lentiviral vector carrying packaging proteins CMVΔR8.2 (Addgene plasmid 12263), and VSV-G were obtained from Addgene.

### Western Blot and Immunoprecipitation

For cell lysate preparation, monolayer cells were lysed with a lysis buffer (50 mM Tris-HCl, pH 7.5, 150 mM NaCl, 0.5% Nonidet P40, 50 mM NaF, 1 mM Na3VO4, 5 mM β-glycerophosphate, 1 mM dithiothreitol, 1 mM phenylmethylsulfonyl fluoride) supplemented with a protease inhibitor mixture (Sigma) on ice. Lysates were cleared by centrifuging at 14,000×g for 20 min. Boiled samples in 2× SDS loading buffer were resolved on a 10–12% SDS-polyacrylamide gel (SDS-PAGE). After electrophoresis, the separated proteins were transferred onto a nitro-cellulose membrane (Bio-Rad). The resulting blots were blocked in 10% milk for 1 h, and then incubated with the rabbit anti-CHIKV E1 serum (1/500) (Santa Cruz Biotechnology, Inc, Santa Cruz, CA) overnight at 4°C. The secondary antibody used in the immunoblot was a 1∶2000 dilution of HRP-linked anti-IgG, followed by detection using the ECL reagents (Amersham).

### Reporter assay

For the luciferase reporter assay, 0.25 million cells/well were seeded in 24-well plates. The next day, cells were transfected with lipofectamine 2000 (Invitrogen Life Technologies). A specified amount of DNA was added into each transfection and an empty pcDNA3.1 plasmid was included when necessary (filler DNA) to keep the total amount of DNA same in each transfection. Cells were harvested between 24 and 48 h after transfection and luciferase activity was measured using the luciferase assay system (Promega). For all experiments, the data presented are the mean ± the standard deviation (SD) of three independent experiments.

### Chikungunya Pseudovirus

Chikungunya pseudovirus was created by transfecting the CHIKV plasmid synthesized by Genscript, ΔR8.2 (HIV Gag-Pol), and pTrip Luciferase. These were all transfected at 1∶1 ratios. After the transfection was completed the cells were allowed to incubate for 24 hours. When 24 hours was over the DMEM complete media was changed with new complete DMEM media. At the end of 48 hours the pseudo-virus was harvested. To harvest the virus the cells and media was spun in a centrifuge at 5000×g for 5 minutes. After 5 minutes the media was removed from the pellet an filtered through a 0.45 µm filter syringe and aliquoted as deemed appropriate and frozen or used immediately for infection. (All controls, such as VSV-G and pcDNA 3.1 blank, were transfected in the same manner). The titer of infectious virus was determined by an end-point assay in which we infected 293T cells with 10-fold serially diluted virus. The dilution that gave 5 fold luciferase activities above background reading is considered positive.

### Viral Infection

Once virus had been harvested after 48 hrs polybrene was added at a concentration of 4 µg/µL. Cell media was removed and the virus containing polybrene was then added to the determined cells. Cells were at a concentration of approximately 70% before they were infected. The virus was left to incubate on the cells in the incubator for 5 hours and was then removed. Fresh pre-warmed complete media was used to replace the virus media.

### Statistical analysis

Student T-tests were performed on all experiments and a p value<0.05 is considered statistically significant.
